# Supplementary material for: Analysis of PPARγ Signaling Activity in Psoriasis
Source: Int J Mol Sci. 2021 Aug 10;22(16):8603. doi: 10.3390/ijms22168603 (PMC8395241; doi:10.3390/ijms22168603)
Supplement: Supplementary file 1 [file ijms-22-08603-s001.zip › Supplemental materials_Analysis of PPARg signaling activity in psoriasis/Pathway models/Models images and html files/Interleukin-17 and interleukin-22 signaling in psoriasis/2896.html]

MAPK14


# Protein MAPK14

|  |  |
| --- | --- |
| URN | urn:agi-llid:1432 |
| Total Entities | 0 |
| Connectivity | 16208 |
| Name | MAPK14 |
| Description | mitogen-activated protein kinase 14 |
| Notes | The protein encoded by this gene is a member of the MAP kinase family. MAP kinases act as an integration point for multiple biochemical signals, and are involved in a wide variety of cellular processes such as proliferation, differentiation, transcription regulation and development. This kinase is activated by various environmental stresses and proinflammatory cytokines. The activation requires its phosphorylation by MAP kinase kinases (MKKs), or its autophosphorylation triggered by the interaction of MAP3K7IP1/TAB1 protein with this kinase. The substrates of this kinase include transcription regulator ATF2, MEF2C, and MAX, cell cycle regulator CDC25B, and tumor suppressor p53, which suggest the roles of this kinase in stress related transcription and cell cycle regulation, as well as in genotoxic stress response. Four alternatively spliced transcript variants of this gene encoding distinct isoforms have been reported. [provided by RefSeq, Jul 2008] |
| Primary Cell Localization | Cytoplasm |
| Class | Protein kinase |
| ObjectType | Protein |

---

|  |  |
| --- | --- |
| Pathway | Alzheimer's Disease |
|  | Osteoarthritis |
|  | Skin Aging |
|  | Oncogene Induced Cellular Senescence |
|  | Dopamine Receptors Signaling |
|  | Serotonin Receptors Signaling |
|  | Thrombopoietin Receptors Signaling in Platelet Maturation |
|  | T-Cell Dependent B-Cell Activation |
|  | T-Cell Maturation (Hypothesis) |
|  | T-Cell Positive Selection and Neglect Induced Death |
|  | T-Cell Central Tolerance |
|  | Th1-Cell Differentiation |
|  | Treg-Cell Differentiation |
|  | Th2-Cell Differentiation |
|  | T-Cell Receptor Signaling |
|  | AHR Signaling in Tr1 Cells Function |
|  | Platelet Activation via Adhesion Molecules |
|  | Scavenger Receptors in Platelet Activation |
|  | Bradykinin Effects in Inflammation |
|  | MC1R in anti-Inflammatory Signaling |
|  | Leukotriene Effect on Vascular Endothelial Cell Response |
|  | Leukotriene Effect on Airway Smooth Muscle Response |
|  | Basophil Activation |
|  | Mast-Cell Activation without Degranulation through TNFSF8 Signaling |
|  | Mast-Cell Activation via IgE Signaling |
|  | Mast-Cell Activation without Degranulation |
|  | Mast-Cell Activation without Degranulation through IL33/IL1RL1 Signaling |
|  | Mast-Cells De Novo Synthesized Mediators via IgE Independent Signaling |
|  | Mast-Cell Activation without Degranulation through IL1R1 and TLR Signaling |
|  | Macrophage M1 Lineage |
|  | Neutrophil Recruitment and Priming |
|  | Neutrophil Degranulation via FPR1 Signaling |
|  | Vascular Smooth Muscle Cell/Pericyte Migration and Proliferation |
|  | Vascular Endothelial Cell Activation by Cytokines |
|  | Vascular Endothelial Cell Activation by Growth Factors |
|  | GFs/TNF -> Ion Channels |
|  | Nociception Expression Targets Signaling |
|  | EDNRA/B -> Vascular Motility |
|  | ADRA1A -> IL6 Production |
|  | ADRA1 -> Prostaglandin Generation |
|  | BDKRB1/2 -> Prostaglandin Generation |
|  | CNR1/2 -> IL1B/2/4/6/10 Production |
|  | DRD2 Expression Targets |
|  | CHRM1 -> IL2 Production |
|  | HTR1 -> IL6 Production |
|  | HTR5 -> TNF Production |
|  | HTR1 -> Membrane Transport |
|  | IL1B Expression Targets -> Nociception |
|  | Luteinizing Hormone in Oocyte Meiotic Resumption |
|  | Circadian Clock Genes in Suprachiasmatic Nuclei Neurons |
|  | Cardiomyocyte Dysfunction |
|  | Proteins Involved in Atherosclerosis |
|  | Scavenger Receptor OLR1 in Inflammation Related Endothelial Dysfunction |
|  | Dendritic Cells Function in Atherosclerosis |
|  | Th1-Cells Activation and Proliferation in Atherosclerosis |
|  | Dilated Cardiomyopathy |
|  | Proteins Involved in Hypertrophic Cardiomyopathy |
|  | Cardiomyocyte Hypertrophy |
|  | Hypertrophic Cardiomyopathy |
|  | Proteins Involved in Arterial Hypertension |
|  | Proteins Involved in Pulmonary Hypertension |
|  | Proteins Involved in Myocardial Ischemia |
|  | Proteins Involved in Myocarditis |
|  | Inflammatory Reaction in Acne Vulgaris |
|  | Keratinocyte Proliferation in Acne Vulgaris |
|  | Atopic Dermatitis Onset |
|  | Mast-Cells Activation in Atopic Dermatitis |
|  | Acute Phase in Atopic Dermatitis |
|  | Atopic Dermatitis |
|  | IL17 Signaling in Psoriasis |
|  | Proteins Involved in Psoriasis |
|  | IL22 Induces Keratinocyte Proliferation in Psoriasis |
|  | Proteins with Altered Expression in Psoriasis |
|  | Dendritic Cells Function in Psoriasis |
|  | Th1-Cell Function in Systemic Lupus Erythematosus |
|  | B-Cell Activation in Systemic Lupus Erythematosus |
|  | T-Cell Cytotoxic Response against Melanocytes in Vitiligo |
|  | Humoral Immunity in Vitiligo |
|  | Treg-Cell Function in Diabetes Mellitus Type 1 |
|  | Natural Killer T-Cell Roles in Diabetes Mellitus Type 1 |
|  | non-Suppressive Treg-Cell in Diabetes Mellitus Type 1 |
|  | TLR2 Signaling in Treg-Cell in Type 1 Diabetes (Animal Model) |
|  | Treg-Cell Activation in Diabetes Mellitus |
|  | Proteins Involved in Diabetes Mellitus Type 2 |
|  | Podocyte Dysfunction in Diabetic Nephropathy |
|  | Proteins Involved in Diabetic Nephropathy |
|  | Mesangial Cell Dysfunction in Diabetic Nephropathy |
|  | Proteins Involved in Diabetic Neuropathy |
|  | Adiponectin in Insulin Resistance Prevention |
|  | Proteins Involved in Insulin Resistance |
|  | Insulin Resistance in Hepatocytes |
|  | Oxidation of Fatty Acid in non-Alcoholic Fatty Liver Disease |
|  | beta-Cell Destruction through Cytokines in Diabetes Mellitus |
|  | Nitric Oxide Effects on beta-Cell |
|  | Immune Sustem Activation in Hashimoto's Thyroiditis |
|  | Hashimoto's Thyroiditis |
|  | Bone Remodeling in Hyperthyroidism |
|  | CD4+T-Cell Response in Celiac Disease |
|  | Celiac Disease |
|  | OipA Signaling in Helicobacter Pylori Infection |
|  | Epithelial Cell in the Innate Immune Response in Ulcerative Colitis |
|  | Th2-Cell Function in Ulcerative Colitis |
|  | Antiphospholipid Antibodies in Platelets and Coagulation Cascade |
|  | Antiphospholipid Antibodies in Endothelial Cell |
|  | Proteins Involved in Antiphospholipid Syndrome |
|  | Proteins Involved in B-Cell Chronic Lymphocytic Leukemia |
|  | Osteoclast Activation in Multiple Myeloma |
|  | Proteins Involved in Multiple Myeloma |
|  | Antiphospholipid Antibodies and Endothelial Cell Interaction |
|  | Platelet and Coagulation Cascade Activation by Antiphospholipid Antibodies |
|  | IL33 Signaling Related Eosinophilia in Asthma |
|  | IgE Induces Airway Smooth Muscle Cell Proliferation |
|  | Basophil Activation in Asthma |
|  | Mast-Cell Activation in Asthma |
|  | Th2-Cell Response in Asthma |
|  | CFTR Facilitates Oxidative Stress in Airway Epithelium in CF |
|  | CFTR Expression in Epithelial Cells (Class I Mutations) |
|  | Osteoblast Function Decline in Gout |
|  | Osteoclast Activation in Gout |
|  | Neutrophil Recruitment in Sinovium in Gout |
|  | Monocytes Function in Gout |
|  | TNF and IL1B Induce Metalloproteinase Synthesis in Osteoarthritis |
|  | Apoptosis of Chondrocyte |
|  | IL1B Induced Arthralgia |
|  | TGFB2 Signaling Impairement in Osteoarthritis |
|  | Synovial Fibroblast Activation in Psoriatic Arthritis |
|  | PTPN22 Role in Rheumatoid Arthritis |
|  | Osteoclast Activation in Rheumatoid Arthritis |
|  | Synovial Fibroblast Activation by Citokines in Rheumatoid Arthritis |
|  | Apoptosis Block in Synovial Fibroblast |
|  | Proteins Involved in Rheumatoid Arthritis |
|  | Autocrine Cytokine/Chemokine Loops in Systemic Scleroderma |
|  | B-Cells Function in Systemic Scleroderma |
|  | Th2-Cells Function in Systemic Scleroderma |
|  | Skin Fibrosis |
|  | CCR5 Signaling in Macrophages |
|  | Apoptosis Block in Infected Cells |
|  | Diabetes Induced Periodontitis |
|  | IL1 Receptor Antagonist (IL1RN) Deficiency (DIRA) |
|  | IL36 Receptor Antagonist (IL1F5) Deficiency (DITRA) |
|  | Cryopyrin (NLPR3) Associated Periodic Syndromes |
|  | Bone Loss in Osteoporosis |
|  | Hyperparathyroidism, Secondary Effect |
|  | Osteoclast Activation in Postmenopause |
|  | Proteins Involved in Alzheimer's Disease |
|  | Glioma Invasion Signaling |
|  | Prostaglandin E2 Synthesis Activation via Glutamate in Familial Hemiplegic Migraine |
|  | Growth Factor Signaling in Neuroblastoma |
|  | Neuroblastoma |
|  | Proteins Involved in Amyotrophic Lateral Sclerosis |
|  | Microglia and Motor Neuron Interaction Dysregulation |
|  | Crystallin alpha B (CRYAB) Suppresses Multiple Sclerosis |
|  | Breast Cancer |
|  | Proteins Involved in Endometriosis |
|  | Angiogenesis in Endometriosis |
|  | Subepithelial Fibroblasts in Middle Ear Cholesteatoma |
|  | HPV E5 Regulates EDNRA and EGFR Signaling Pathway in Keratinocytes |
|  | Steroids Induced Cataract |
|  | VEGFA in Neovascular Glaucoma |
|  | Neurotrophic Factor Deprivation in Retinal Ganglion Cell Death |
|  | Cytokines Trigger Otitis Media |
|  | MUC5AC Expression by NTHi and S. Pneumoniae |
|  | Proteins Involved in Glomerulonephritis |
|  | Endothelial Cell Dysfunction in Glomerulonephritis |
|  | Podocyte Dysfunction in Glomerulonephritis |
|  | Mesangial Cell Dysfunction in Glomerulonephritis |
|  | Interstitial Fibroblasts in Pyelonephritis |
|  | Endothelial Cell Dysfunction in Pyelonephritis |
|  | ANGPT1 -> CREB/CREBBP Expression Targets |
|  | ANGPT2 -> AP-1/CREBBP/MYC Expression Targets |
|  | AREG -> NFATC Expression Target |
|  | AREG -> CREB/CREBBP Expression Targets |
|  | AREG -> AP-1 Expression Targets |
|  | Acetylcholine Expression Targets |
|  | Dopamine/Gi Expression Targets |
|  | BTC -> NFATC Expression Targets |
|  | BTC -> AP-1/ATF/CREB Expression Targets |
|  | CXCR4 -> ARRB1 Signaling |
|  | Sialophorin -> CTNNB/MYC/TP53 Signaling |
|  | CD72 -> NF-kB Expression Targets |
|  | CD8 -> NF-kB Expression Targets |
|  | CD80 -> NFATC Expression Targets |
|  | CD86 -> NFATC Expression Targets |
|  | CCL3 Expression Targets |
|  | CCL7 Expression Target |
|  | IL8 Expression Targets |
|  | Complement Component GPCRs Signaling |
|  | Doxorubicin Induced Cardiotoxicity |
|  | Clozapine Induced Granulocytopenia |
|  | Cyclosporine Induced Nephrotoxicity |
|  | EGF -> AP-1/ATF Expression Targets |
|  | EGF -> CREB/CREBBP/ELK/SRF/MYC Expression Targets |
|  | EGF -> MEF/MYOD/NFATC Expression Targets |
|  | EGF -> TP53 Expression Targets |
|  | EGFR/ERBB3 -> MEF/MYOD/NFATC/MYOG Signaling |
|  | EGFR/ERBB2 -> TP53 Signaling |
|  | EGFR -> AP-1/ATF2 Signaling |
|  | EGFR -> AP-1/CREB/ELK/SRF/MYC Signaling |
|  | EREG -> CREB Expression Target |
|  | EREG -> AP-1/ATF Expression Targets |
|  | Erythropoietin -> AP-1/MYC/CREB Expression Targets |
|  | F2 -> AP-1/CREB/ELK/SRF/SP1 Expression Targets |
|  | PLG -> AP-1/CREB/ELK/SRF/SP1 Expression Targets |
|  | FGF9 -> AP-1/CREB/MYC Expression Targets |
|  | FGF10 -> AP-1/CREB/CREBBP/MYC Expression Targets |
|  | FGF1 -> AP-1/CREB/ELK/SRF/MYC Expression Targets |
|  | FGF8 -> AP-1/CREB/MYC Expression Targets |
|  | FGF18 -> AP-1/CREB Expression Targets |
|  | FGF4 -> AP-1/MYC Expression Targets |
|  | FGF7 -> AP-1/CREB/CREBBP/MYC Expression Targets |
|  | FGF2 -> AP-1/CREB/CREBBP/ELK/SRF/MYC Expression Targets |
|  | FGFR -> AP-1/CREB/CREBBP/ELK/SRF/MYC Signaling |
|  | ADCYAP1 Expression Targets |
|  | Glutamate/Gq Expression Targets |
|  | VIP Expression Targets |
|  | HBEGF -> AP-1/ATF Expression Targets |
|  | HBEGF -> TP53 Expression Targets |
|  | HBEGF -> MEF/MYOD Expression Target |
|  | HBEGF -> CREB/MYC Expression Targets |
|  | HGF -> AP-1/CREB/ELK/SRF/MYC Expression Targets |
|  | HGFR -> AP-1/CREB/MYC Signaling |
|  | RAS/RAF/MAPK Signaling Activation in Cancer |
|  | RAS/RAF/MAPK Signaling Activation by Blocking of Tumor Suppressors |
|  | TGFB Signaling activation by Blocking of Tumor Suppressors |
|  | Oxidized LDL/OLR1 Activates Cell Proliferation in Cancer |
|  | TGFB Family in Epithelial to Mesenchymal Transition in Cancer |
|  | IGF1 -> MEF/MYOD/MYOG Expression Targets |
|  | IGF2/MEF/MYOD Expression Targets |
|  | Insulin -> MEF/MYOD Expression Targets |
|  | ICAM1 -> AP-1/CREB/ELK/SRF Signaling |
|  | IGF1R -> MEF/MYOD/MYOG Signaling |
|  | FibronectinR -> NF-kB Signaling |
|  | FibronectinR -> AP-1/ELK/SRF/SREBF Signaling |
|  | Fibronectin Expression Targets |
|  | CTGF -> AP-1/CREB/MYC Expression Targets |
|  | ICAM1 Expression Targets |
|  | IL1R -> NF-kB Signaling |
|  | IL16-> ATF/CREB/CREBBP Expression Target |
|  | PAF/Gq -> AP-1/ATF1/CREB/ERK/SRF Expression Targets |
|  | Dronabinol/Anandamide Expression Targets |
|  | PAF/Gq -> NF-kB Expression Targets |
|  | S1P Expression Targets |
|  | NRG1 -> MEF/MYOD Expression Targets |
|  | NRG1 -> CREB/CREBBP/ELK/SRF/MYC Expression Targets |
|  | NRG1 -> TP53 Expression Target |
|  | NRG1 -> AP-1/ATF Expression Targets |
|  | NGF -> SMAD3/NF-kB Expression Targets |
|  | NGF -> AP-1/TP53/MYC Expression Targets |
|  | NGF -> FOXO/MYCN/ELK-SRF Expression Targets |
|  | BDNF Expression Targets |
|  | NTF3 Expression Targets |
|  | NTF4 Expression Targets |
|  | NGF -> CREB/CEBPB/MEF2A Expression Targets |
|  | NOTCH -> TCF3 Signaling |
|  | NOTCH Expression Targets |
|  | PDGFB -> AP-1/CREB/MYC Expression Target |
|  | PDGFC -> CREB Expression Target |
|  | PDGFD -> AP-1 Expression Targets |
|  | PDGF -> AP-1/CREB/CREBBP/MYC Expression Targets |
|  | PDGFR -> AP-1/MYC Signaling |
|  | AVP/Gs -> CREB/ELK/SRF/AP-1/EGR Expression Targets |
|  | AGT -> TP53 Expression Targets |
|  | EDN1 Expression Targets |
|  | AGT -> CREB Expression Targets |
|  | AVP/Gs -> MEF/MYOD/NFATC/MYOG Expression Targets |
|  | AGT -> ELK/SRF Expression Targets |
|  | AVP/Gq -> MEF/MYOD/NFATC/MYOG Expression Targets |
|  | EDN3 Expression Targets |
|  | AVP/Gq -> CREB/ELK/SRF/AP-1/EGR Expression Targets |
|  | PDCD1 -> ATF/CREB/CREBBP Expression Targets |
|  | DDR1 -> NF-kB Signaling |
|  | AngiopoietinR -> AP-1 Signaling |
|  | NTRK -> AP-1/CREB/ELK/SRF/MYC/SMAD3/TP53 Signaling |
|  | GAS6 -> AP-1/CREB Expression Targets |
|  | FIGF -> AP-1 Expression Target |
|  | KITLG -> AP-1/CREB/CREBBP/MYC Expression Targets |
|  | Collagen -> NF-kB Expression Targets |
|  | CSF1 -> AP-1/CREB/CREBBP/MYC Expression Targets |
|  | PGF -> AP-1/CREB/CREBBP/MYC Expression Targets |
|  | FLT3LG -> AP-1/CREB/CREBBP Expression Targets |
|  | TGFBR -> AP-1 Signaling |
|  | TGFBR -> CREB/ELK-SRF Signaling |
|  | TGFBR -> ATF/GADD/MAX/TP53 Signaling |
|  | TGFBR -> MEF/MYOD/MYOG Signaling |
|  | TNF-alpha/TNFRSF1A Signaling |
|  | TGF-beta Signaling |
|  | BMPR2 Signaling |
|  | P38 MAPK/MAPK14 Signaling |
|  | AXL Receptor Tyrosine Kinase Signaling |
|  | PI3K/RAC1 Signaling |
|  | TNF-alpha/TNFRSF1B Signaling |
|  | KRAS Signaling |
|  | VEGF Signaling |
|  | TCR -> CREB/CREBBP/ATF Expression Targets |
|  | T-Cell Receptor -> NFATC Signaling |
|  | T-Cell Receptor -> ATF/CREB Signaling |
|  | TGFA -> TP53 Expression Targets |
|  | TGFA -> MEF/MYOD/NFATC Expression Targets |
|  | TGFA/AP-1/ATF Expression Targets |
|  | TGFA -> CREB/CREBBP/ELK-SRF/MYC Expression Targets |
|  | TGFB3-TGFBR2 Expression Targets |
|  | TGFB2-TGFBR2 Expression Targets |
|  | TGFB1-TGFBR2 Expression Targets |
|  | FASLG Expression Targets |
|  | TNF -> NF-kB Expression Targets |
|  | TNFSF10 Expression Targets |
|  | LTA Expression Targets |
|  | TNFSF14 Expression Targets |
|  | TNF -> AP-1 Expression Targets |
|  | CD72 -> AP-1 Expression Targets |
|  | CD40LG -> NF-kB/ELK/SRF -> CREB/NFATC Expression Targets |
|  | TNFSF13 Expression Targets |
|  | TNFSF13B Expression Targets |
|  | TNF -> ELK-SRF Expression Targets |
|  | TNF -> CREB Expression Targets |
|  | TNF -> TP53/ATF Expression Targets |
|  | EDA Expression Targets |
|  | EctodysplasinR -> NF-kB Signaling |
|  | TNFR -> CREB/ELK-SRF Signaling |
|  | TNFR -> AP-1/ATF/TP53 Signaling |
|  | NGFR -> AP-1/CEBPB/CREB/ELK/SRF/TP53 Signaling |
|  | TNFRSF5/6 -> RB1/E2F Signaling |
|  | NGFR -> NF-kB Signaling |
|  | TNFR -> NF-kB Signaling |
|  | NGFR -> MEF Signaling |
|  | TNFRSF6 -> DDIT3 Signaling |
|  | TNFRSF1A -> AP-1/ATF/TP53 Signaling |
|  | TNFRSF1A -> CREB/ELK-SRF Signaling |
|  | Thrombopoietin -> AP-1/CREB/CREBBP/MYC Expression Targets |
|  | TLR4 -> AP-1 Expression Targets |
|  | TLR5 Expression Targets |
|  | TLR7 Expression Targets |
|  | TLR4 -> AP-1/EGR1/HIF1A Expression Targets |
|  | TLR1 -> 2/6 Expression Targets |
|  | IL1B Expression Targets |
|  | IL1A Expression Targets |
|  | TLR9 Expression Targets |
|  | TLR -> AP-1 Signaling |
|  | ER Stress (Unfolded Protein Response) |
|  | ROS in Angiotensin Mediated Cardiovascular Remodeling and Hypertrophy |
|  | VEGFA -> ATF/CREB/ELK-SRF Expression Targets |
|  | VEGFA -> NFATC Expression Targets |
|  | VEGFC -> ATF Expression Target |
|  | VEGFA -> AP-1/CREBBP/MYC Expression Targets |
|  | VEGFR -> NFATC Signaling |
|  | VEGFR -> AP-1/CREB/MYC Signaling |
|  | VEGFR -> ATF/CREB/ELK-SRF Signaling |
|  | pathway 3 |
|  | e |
|  | pathway 4 |
|  | ace2 |
|  | New Pathway (2) |
|  | cypa |
|  | sars-cov no binding |
|  | dsRNA induces innate immunity and INFs in alveolar cells |
|  | 1\_Inflammation in keratinocytes in the pathology of acne |
|  | 2\_Interleukin-17 and interleukin-22 signaling in psoriasis |
|  | 1\_The onset of atopic dermatitis |
|  | 1\_TNF and IL1B provoke ECM degradation in osteoarthritis |
|  | 1\_Osteoclast-mediated bone resorption in postmenopausal women |
|  | 1\_Osteoclast functional impairment in osteopetrosis |
|  | 2\_Chondrocyte death in osteoarthritis |
|  | 2\_The role of endothelial cells in pyelonephritis |
|  | 3\_The role of interstitial fibroblasts in pyelonephritis |
|  | 3\_Mesangial cell dysfunction in glomerulonephritis |
|  | 2\_Podocyte dysfunction in glomerulonephritis |
|  | 3\_Role of neurotrophic factor deprivation in retinal ganglion cell death |
|  | 2\_Pathogens stimulate mucins expression in the middle ear |
|  | 2\_Oxidized LDL causes endothelial dysfunction in atherosclerosis |
|  | 2\_Cardiomyocyte hypertrophy |
|  | 3\_Cardiomyocyte dysfunction unrelated to cell junctions in ARVD |
|  | 4\_Inflammatory mediators inhibit GATA signaling in erythropoiesis |
|  | 4\_2\_Insulin resistance: inflammation-related insulin resistance |
|  | 4\_3\_Insulin resistance: FFA-related insulin resistance |
|  | drug-target moltransport |
|  | DIPG overview\_1 (histones) |
|  | New Pathway (1) |
|  | mapk1 |
|  | New Pathway |
|  | mapk1 protein modificators |
|  | Interleukin-17 and interleukin-22 signaling in psoriasis |

---

|  |  |
| --- | --- |
| Group | other partners for SARS |

---

|  |  |
| --- | --- |
| Source | curated |
|  | ResNet |
|  | GO component |
|  | Biopax |

---

|  |  |
| --- | --- |
| MedScan ID | 1432 |

---

|  |  |
| --- | --- |
| LocusLink ID | 1432 |
|  | 26416 |
|  | 81649 |
|  | 12975 |
|  | 326416 |
|  | 282273 |
|  | 534492 |
|  | 65237 |
|  | 321438 |
|  | 336914 |

---

|  |  |
| --- | --- |
| Alias | Csaids-binding protein |
|  | mitogen-activated protein kinase p38 alpha |
|  | mitogen-activated protein kinase p38 |
|  | tRNA synthetase cofactor p38 |
|  | cytokine suppressive anti-inflammatory drug-binding protein |
|  | LOC101929346 |
|  | p38/Mpk2 |
|  | MAPK p38alpha |
|  | p38alpha Exip |
|  | cytokine suppressive anti-inflammatory drug binding protein |
|  | MAX-interacting protein II |
|  | CSBP2 |
|  | PRKM15 |
|  | MAX-interactive protein 2 |
|  | CSBP/p38 MAPK |
|  | CSBP/p38 MAP kinase |
|  | cytokine-supressive anti-inflammatory drug-binding protein |
|  | CSAID-binding protein |
|  | OTTMUSP00000021707 |
|  | MAP kinase p38 alpha |
|  | Mpk2/p38 |
|  | RP24-137E7.1 |
|  | CSPB I |
|  | p38 mitogen activated protein kinase |
|  | CSBP/p38 kinase |
|  | MAP kinase p38 |
|  | p38ALPHA |
|  | p38alpha mitogen-activated protein kinase |
|  | Crk1 |
|  | MAX-interacting protein 2 |
|  | CSBP |
|  | Exip |
|  | MAP kinase MXI2 |
|  | CSPB1 |
|  | MAP kinase 14 |
|  | RP1-179N16.5 |
|  | PRKM14 |
|  | p38 SAPK |
|  | mitogen-activated protein kinase 14A |
|  | MGC105413 |
|  | OTTMUSP00000021708 |
|  | MAX-interactive protein II |
|  | p38 MAP kinase |
|  | Mxi2 |
|  | stress-activated protein kinase p38 |
|  | p38Hog |
|  | OTTMUSP00000021706 |
|  | p38a |
|  | p38 MAP kinase alpha |
|  | CSBP1 |
|  | OTTHUMP00000216566 |
|  | OTTHUMP00000216563 |
|  | Csaids binding protein |
|  | MAPK14 |
|  | mitogen activated protein kinase 14 |
|  | MGC102436 |
|  | cytokine suppressive anti-inflammatory drug binding protein 1 |
|  | tRNA synthase cofactor p38 |
|  | cytokine-supressive anti-inflammatory drug binding protein |
|  | CSBP I |
|  | cytokine suppressive anti-inflammatory drug binding protein I |
|  | p38MAPK |
|  | CSAID binding protein |
|  | MAPK p38 |
|  | p38alpha MAPK |
|  | OTTMUSP00000021709 |
|  | p38alpha MAP kinase |
|  | SAPK2A |
|  | p38 kinase |
|  | stress-activated protein kinase 2A |
|  | RK |
|  | p38 |
|  | mitogen-activated protein kinase 14 |
|  | p38-alpha |
|  | MAPK 14 |
|  | p38 MAPK |
|  | Hog |
|  | MAP kinase 2 |
|  | reactive kinase |
|  | p38 alpha |
|  | Mitogen-activated protein kinase p38 |
|  | PRKM14s |
|  | PRKM15s |
|  | MAPK14s |
|  | Lil I |
|  | Serum cholesterol level QTL XII |
|  | Serum cholesterol level QTL 12 |
|  | P-38 |
|  | Lil1 |
|  | Lipid Level QTL 1 |
|  | Scl12 |
|  | Lipid Level QTL I |

---

|  |  |
| --- | --- |
| EC Number | 2.7.11.24 |
|  | 2.7.1.37 |
|  | 2.7.1.- |
|  | 2.7.11.1 |

---

|  |  |
| --- | --- |
| GO ID | 0005524 |
|  | 0004707 |
|  | 0004708 |
|  | 0051525 |
|  | 0019899 |
|  | 0048273 |
|  | 0019903 |
|  | 0004674 |
|  | 0070935 |
|  | 0000077 |
|  | 0007265 |
|  | 0000187 |
|  | 0001525 |
|  | 0006915 |
|  | 0001502 |
|  | 0000902 |
|  | 0007166 |
|  | 0071479 |
|  | 0071222 |
|  | 0071223 |
|  | 0071310 |
|  | 0071356 |
|  | 0035924 |
|  | 0098586 |
|  | 0006935 |
|  | 0002062 |
|  | 0019395 |
|  | 0006006 |
|  | 0035556 |
|  | 0031663 |
|  | 0090090 |
|  | 0043312 |
|  | 0030316 |
|  | 0038066 |
|  | 0018105 |
|  | 0001890 |
|  | 0043536 |
|  | 0090336 |
|  | 0060045 |
|  | 0031281 |
|  | 0002741 |
|  | 0045648 |
|  | 0010628 |
|  | 0046326 |
|  | 2001184 |
|  | 0010759 |
|  | 1905050 |
|  | 0051149 |
|  | 0045663 |
|  | 1901741 |
|  | 0010831 |
|  | 0042307 |
|  | 2000379 |
|  | 0045944 |
|  | 0051090 |
|  | 1900015 |
|  | 0010468 |
|  | 0030278 |
|  | 1901796 |
|  | 0099179 |
|  | 0006357 |
|  | 0032495 |
|  | 0035994 |
|  | 0007165 |
|  | 0042770 |
|  | 0007519 |
|  | 0090400 |
|  | 0051146 |
|  | 0007178 |
|  | 0048010 |
|  | 0005737 |
|  | 0005829 |
|  | 0005576 |
|  | 1904813 |
|  | 0098978 |
|  | 0005739 |
|  | 0016607 |
|  | 0005654 |
|  | 0005634 |
|  | 0034774 |
|  | 0000922 |
|  | 0016301 |
|  | 0000166 |
|  | 0008022 |
|  | 0004672 |
|  | 0016740 |
|  | 0006974 |
|  | 0014835 |
|  | 0016310 |
|  | 0046777 |
|  | 0006468 |
|  | 0006355 |
|  | 0032496 |
|  | 0051403 |
|  | 0005623 |
|  | 0009749 |
|  | 0005622 |
|  | 0045087 |
|  | 0002756 |
|  | 0002755 |
|  | 0016071 |
|  | 0007243 |
|  | 0016070 |
|  | 0007005 |
|  | 0006950 |
|  | 0005515 |
|  | 0071363 |
|  | 0035666 |
|  | 0008063 |
|  | 0044445 |
|  | 0070062 |
|  | 0048011 |
|  | 0034166 |
|  | 0038123 |
|  | 0038124 |
|  | 0034162 |
|  | 0007596 |
|  | 0002224 |
|  | 0000165 |
|  | 0006996 |
|  | 0034146 |
|  | 0034142 |
|  | 0006351 |
|  | 0042692 |
|  | 0023014 |
|  | 0006928 |
|  | 0010467 |
|  | 0034134 |
|  | 0034138 |
|  | 0034130 |
|  | 0030168 |
|  | 0016772 |
|  | 0008152 |
|  | 0005625 |
|  | 0008339 |
|  | 0004682 |
|  | 0006960 |
|  | 0004713 |
|  | 0019735 |
|  | 0004691 |
|  | 0040016 |

---

|  |  |
| --- | --- |
| KEGG ID | hsa:1432 |
|  | mmu:26416 |
|  | rno:81649 |

---

|  |  |
| --- | --- |
| MedLine Reference | 10601328 |
|  | 11374881 |
|  | 11466386 |
|  | 11687663 |
|  | 11790788 |
|  | 11827958 |
|  | 11884615 |
|  | 11925444 |
|  | 11989977 |
|  | 11994045 |
|  | 12076985 |
|  | 12076986 |
|  | 12087100 |
|  | 12091394 |
|  | 12096921 |
|  | 12132588 |
|  | 12181122 |
|  | 12225963 |
|  | 12297293 |
|  | 12324467 |
|  | 12349907 |
|  | 12369731 |
|  | 12399465 |
|  | 12403788 |
|  | 12409219 |
|  | 12409227 |
|  | 12414803 |
|  | 12419320 |
|  | 12444545 |
|  | 12446726 |
|  | 12477932 |
|  | 12538734 |
|  | 12558602 |
|  | 12564765 |
|  | 12606350 |
|  | 12609991 |
|  | 12637564 |
|  | 12700162 |
|  | 12724128 |
|  | 12744493 |
|  | 12800247 |
|  | 12808350 |
|  | 12837681 |
|  | 12842450 |
|  | 12849998 |
|  | 12869527 |
|  | 12876381 |
|  | 12881212 |
|  | 12883833 |
|  | 12928445 |
|  | 12944406 |
|  | 12952860 |
|  | 12957486 |
|  | 12970901 |
|  | 14522818 |
|  | 14530261 |
|  | 14593093 |
|  | 14593211 |
|  | 14602083 |
|  | 14606087 |
|  | 14617573 |
|  | 14631143 |
|  | 14633134 |
|  | 14645111 |
|  | 14648549 |
|  | 14660488 |
|  | 14749328 |
|  | 14752292 |
|  | 14762791 |
|  | 14764603 |
|  | 14962831 |
|  | 14970194 |
|  | 14988658 |
|  | 15001568 |
|  | 15007040 |
|  | 15051668 |
|  | 15051723 |
|  | 15081310 |
|  | 15084475 |
|  | 15167682 |
|  | 15220210 |
|  | 15271927 |
|  | 15297444 |
|  | 15309413 |
|  | 15365619 |
|  | 15465893 |
|  | 15489642 |
|  | 15539417 |
|  | 15545827 |
|  | 15590660 |
|  | 15591055 |
|  | 15617763 |
|  | 15634764 |
|  | 15647278 |
|  | 15649892 |
|  | 15650391 |
|  | 15664670 |
|  | 15721267 |
|  | 15748291 |
|  | 15755725 |
|  | 15826721 |
|  | 15831522 |
|  | 15857906 |
|  | 15883033 |
|  | 15896702 |
|  | 15948190 |
|  | 15948714 |
|  | 15994754 |
|  | 16014723 |
|  | 16037234 |
|  | 16040713 |
|  | 16043884 |
|  | 16118209 |
|  | 16123104 |
|  | 16157584 |
|  | 16183734 |
|  | 16210322 |
|  | 16246848 |
|  | 16247337 |
|  | 16284105 |
|  | 16316999 |
|  | 16326014 |
|  | 16365184 |
|  | 16407200 |
|  | 16409471 |
|  | 16469826 |
|  | 16495005 |
|  | 16531007 |
|  | 16542681 |
|  | 16575906 |
|  | 16751295 |
|  | 16782845 |
|  | 16813528 |
|  | 16816533 |
|  | 16818494 |
|  | 16870744 |
|  | 16891390 |
|  | 16893669 |
|  | 16895802 |
|  | 16911916 |
|  | 16978658 |
|  | 17034759 |
|  | 17161379 |
|  | 70317 |
|  | 7914033 |
|  | 9513091 |
|  | 9779826 |
|  | 10066767 |
|  | 10349636 |
|  | 10629051 |
|  | 10704466 |
|  | 10922068 |
|  | 10943842 |
|  | 10944112 |
|  | 10949032 |
|  | 10973481 |
|  | 11042159 |
|  | 11076861 |
|  | 11217851 |
|  | 11279073 |
|  | 11490018 |
|  | 11744690 |
|  | 11756692 |
|  | 11792567 |
|  | 11792570 |
|  | 11792706 |
|  | 11796722 |
|  | 11880364 |
|  | 11937560 |
|  | 11948180 |
|  | 11960370 |
|  | 11997522 |
|  | 12015964 |
|  | 12021326 |
|  | 12032842 |
|  | 12055252 |
|  | 12058061 |
|  | 12060739 |
|  | 12068299 |
|  | 12082638 |
|  | 12085353 |
|  | 12096337 |
|  | 12112010 |
|  | 12138127 |
|  | 12149242 |
|  | 12175822 |
|  | 12202685 |
|  | 12223406 |
|  | 12232043 |
|  | 12242275 |
|  | 12244175 |
|  | 12359721 |
|  | 12359878 |
|  | 12369781 |
|  | 12370805 |
|  | 12374793 |
|  | 12384504 |
|  | 12386165 |
|  | 12391245 |
|  | 12393706 |
|  | 12393894 |
|  | 12414118 |
|  | 12421981 |
|  | 12425949 |
|  | 12466851 |
|  | 12478662 |
|  | 12482988 |
|  | 12506138 |
|  | 12581156 |
|  | 12589053 |
|  | 12593847 |
|  | 12612065 |
|  | 12637498 |
|  | 12695487 |
|  | 12697715 |
|  | 12717433 |
|  | 12719557 |
|  | 12750397 |
|  | 12788225 |
|  | 12802337 |
|  | 12805214 |
|  | 12808090 |
|  | 12819782 |
|  | 12855555 |
|  | 12893778 |
|  | 12897243 |
|  | 12937170 |
|  | 12947324 |
|  | 12951358 |
|  | 12960069 |
|  | 14499647 |
|  | 14561730 |
|  | 14578350 |
|  | 14585994 |
|  | 1459009 |
|  | 14592977 |
|  | 14593000 |
|  | 14606520 |
|  | 14617800 |
|  | 14622137 |
|  | 14626445 |
|  | 14634061 |
|  | 14665434 |
|  | 14699442 |
|  | 14712227 |
|  | 14718543 |
|  | 14763996 |
|  | 14767066 |
|  | 14966273 |
|  | 14978104 |
|  | 14985505 |
|  | 14991053 |
|  | 15024092 |
|  | 15031106 |
|  | 15102471 |
|  | 15107845 |
|  | 15143069 |
|  | 15159407 |
|  | 15184078 |
|  | 15192112 |
|  | 15195698 |
|  | 15232685 |
|  | 15240007 |
|  | 15282297 |
|  | 15287722 |
|  | 15289440 |
|  | 15316018 |
|  | 15319438 |
|  | 15345139 |
|  | 15381256 |
|  | 15385464 |
|  | 15388329 |
|  | 15459198 |
|  | 15483225 |
|  | 15485852 |
|  | 15509711 |
|  | 15523637 |
|  | 15537893 |
|  | 15557738 |
|  | 15561967 |
|  | 15567067 |
|  | 15572149 |
|  | 15572667 |
|  | 15590669 |
|  | 15601830 |
|  | 15611087 |
|  | 15625301 |
|  | 15630166 |
|  | 15632024 |
|  | 15648447 |
|  | 15649893 |
|  | 15652346 |
|  | 15661878 |
|  | 15687248 |
|  | 15691843 |
|  | 15708845 |
|  | 15735648 |
|  | 15746179 |
|  | 15760340 |
|  | 15761494 |
|  | 15761973 |
|  | 15764603 |
|  | 15767263 |
|  | 15767678 |
|  | 15772134 |
|  | 15778378 |
|  | 15778394 |
|  | 15782199 |
|  | 15795936 |
|  | 15808838 |
|  | 15817653 |
|  | 15824134 |
|  | 15833736 |
|  | 15836771 |
|  | 15837794 |
|  | 15850458 |
|  | 15864310 |
|  | 15870258 |
|  | 15890335 |
|  | 15893881 |
|  | 15905332 |
|  | 15907784 |
|  | 15910777 |
|  | 15919053 |
|  | 15923604 |
|  | 15936714 |
|  | 15946254 |
|  | 15955110 |
|  | 15964803 |
|  | 15971269 |
|  | 15979729 |
|  | 16020390 |
|  | 16098594 |
|  | 16116476 |
|  | 16120811 |
|  | 16135819 |
|  | 16138010 |
|  | 16141072 |
|  | 16141073 |
|  | 16143643 |
|  | 16156785 |
|  | 16169504 |
|  | 16207331 |
|  | 16217033 |
|  | 16272151 |
|  | 16275897 |
|  | 16288922 |
|  | 16289764 |
|  | 16297369 |
|  | 16306704 |
|  | 16357362 |
|  | 16364914 |
|  | 16387856 |
|  | 16400028 |
|  | 16443758 |
|  | 16481366 |
|  | 16492136 |
|  | 16507991 |
|  | 16547729 |
|  | 16571868 |
|  | 16585552 |
|  | 16595708 |
|  | 16603589 |
|  | 16616932 |
|  | 16632522 |
|  | 16650898 |
|  | 16699960 |
|  | 16714293 |
|  | 16751104 |
|  | 16775014 |
|  | 16849316 |
|  | 16849392 |
|  | 16880512 |
|  | 16887817 |
|  | 16912279 |
|  | 16920928 |
|  | 16935932 |
|  | 16936205 |
|  | 16966377 |
|  | 16979163 |
|  | 17015449 |
|  | 17028194 |
|  | 17030606 |
|  | 17052691 |
|  | 17071578 |
|  | 17074887 |
|  | 17077290 |
|  | 17083486 |
|  | 17088247 |
|  | 17090686 |
|  | 17105401 |
|  | 17105766 |
|  | 17139251 |
|  | 17214902 |
|  | 8444355 |
|  | 8449498 |
|  | 8925912 |
|  | 9806642 |
|  | 10085140 |
|  | 10206983 |
|  | 10330143 |
|  | 10348343 |
|  | 10364159 |
|  | 10391943 |
|  | 10415025 |
|  | 10557092 |
|  | 10559944 |
|  | 10581258 |
|  | 10597297 |
|  | 10644717 |
|  | 10706854 |
|  | 10727080 |
|  | 10747897 |
|  | 10779345 |
|  | 10805738 |
|  | 10806207 |
|  | 10838079 |
|  | 10849446 |
|  | 10857861 |
|  | 1088347 |
|  | 10884347 |
|  | 10910078 |
|  | 10912793 |
|  | 10942774 |
|  | 10961885 |
|  | 10978317 |
|  | 10980603 |
|  | 11042204 |
|  | 11042694 |
|  | 11157753 |
|  | 11226159 |
|  | 11259586 |
|  | 11333986 |
|  | 11344273 |
|  | 11346645 |
|  | 11359773 |
|  | 11377386 |
|  | 11387337 |
|  | 11390380 |
|  | 11416124 |
|  | 11489891 |
|  | 11495898 |
|  | 11551930 |
|  | 11551945 |
|  | 11602244 |
|  | 11604491 |
|  | 11691836 |
|  | 11741533 |
|  | 11756422 |
|  | 11773065 |
|  | 11777913 |
|  | 11781324 |
|  | 11788583 |
|  | 11788789 |
|  | 11847341 |
|  | 11848686 |
|  | 11852102 |
|  | 11866441 |
|  | 11896401 |
|  | 11994493 |
|  | 12009331 |
|  | 12051955 |
|  | 12067303 |
|  | 12071847 |
|  | 12085235 |
|  | 12105188 |
|  | 12110590 |
|  | 12126643 |
|  | 12138194 |
|  | 12164931 |
|  | 12170268 |
|  | 12171600 |
|  | 12183063 |
|  | 12200131 |
|  | 12225289 |
|  | 12296996 |
|  | 12370831 |
|  | 12391307 |
|  | 12429732 |
|  | 12473116 |
|  | 12505871 |
|  | 12509443 |
|  | 12519482 |
|  | 12589052 |
|  | 12595530 |
|  | 12606502 |
|  | 12631076 |
|  | 12654092 |
|  | 12663671 |
|  | 12665511 |
|  | 12665801 |
|  | 12684028 |
|  | 12697810 |
|  | 12698197 |
|  | 12702497 |
|  | 12761180 |
|  | 12784337 |
|  | 12810082 |
|  | 12855667 |
|  | 12882963 |
|  | 12897141 |
|  | 12933809 |
|  | 13679851 |
|  | 14512875 |
|  | 14552880 |
|  | 14586399 |
|  | 14648593 |
|  | 14714552 |
|  | 14715645 |
|  | 14970175 |
|  | 14989597 |
|  | 15132952 |
|  | 15133024 |
|  | 15170913 |
|  | 15245437 |
|  | 15246972 |
|  | 15256218 |
|  | 15257094 |
|  | 15273249 |
|  | 15284239 |
|  | 15292176 |
|  | 15292225 |
|  | 15304097 |
|  | 15304486 |
|  | 15310753 |
|  | 15315972 |
|  | 15322009 |
|  | 15345584 |
|  | 15375157 |
|  | 15383283 |
|  | 15388348 |
|  | 15489375 |
|  | 15514847 |
|  | 15516490 |
|  | 15527495 |
|  | 15541600 |
|  | 15542843 |
|  | 15550393 |
|  | 15569672 |
|  | 15578697 |
|  | 15592496 |
|  | 15625302 |
|  | 15629715 |
|  | 15632082 |
|  | 15650392 |
|  | 15665518 |
|  | 15668322 |
|  | 15677464 |
|  | 15728454 |
|  | 15737629 |
|  | 15737736 |
|  | 15753227 |
|  | 15778365 |
|  | 15784503 |
|  | 15790570 |
|  | 15791651 |
|  | 15797859 |
|  | 15843433 |
|  | 15845648 |
|  | 15849811 |
|  | 15851480 |
|  | 15862947 |
|  | 15894558 |
|  | 15901830 |
|  | 15917247 |
|  | 15946948 |
|  | 15950779 |
|  | 15964514 |
|  | 16027723 |
|  | 16027724 |
|  | 16098034 |
|  | 16099944 |
|  | 16123320 |
|  | 16133687 |
|  | 16135815 |
|  | 16138080 |
|  | 16139274 |
|  | 16153249 |
|  | 16153436 |
|  | 16164755 |
|  | 16189514 |
|  | 16197369 |
|  | 16214108 |
|  | 16242072 |
|  | 16251188 |
|  | 16256071 |
|  | 16256948 |
|  | 16257181 |
|  | 16278378 |
|  | 16308312 |
|  | 16338464 |
|  | 16342939 |
|  | 16344056 |
|  | 16351573 |
|  | 16380078 |
|  | 16410245 |
|  | 16410316 |
|  | 16446363 |
|  | 16467496 |
|  | 16469115 |
|  | 16478922 |
|  | 16516911 |
|  | 16538384 |
|  | 16554354 |
|  | 16581800 |
|  | 16613612 |
|  | 16627995 |
|  | 16636664 |
|  | 16645187 |
|  | 16648477 |
|  | 16648633 |
|  | 16683917 |
|  | 16697418 |
|  | 16699462 |
|  | 16699726 |
|  | 16709866 |
|  | 16720051 |
|  | 16769768 |
|  | 16790501 |
|  | 16820791 |
|  | 16843435 |
|  | 16861915 |
|  | 16864945 |
|  | 16869889 |
|  | 16899715 |
|  | 16912047 |
|  | 16924420 |
|  | 16964394 |
|  | 16973972 |
|  | 17030506 |
|  | 17030510 |
|  | 17035228 |
|  | 17050539 |
|  | 17054722 |
|  | 17055984 |
|  | 17059827 |
|  | 17107667 |
|  | 17141198 |
|  | 17142767 |
|  | 7479834 |
|  | 7493921 |
|  | 7535770 |
|  | 7592979 |
|  | 7696354 |
|  | 7716521 |
|  | 7750576 |
|  | 7839144 |
|  | 7997261 |
|  | 8083223 |
|  | 8274451 |
|  | 8530093 |
|  | 8548291 |
|  | 8622669 |
|  | 8626699 |
|  | 8650547 |
|  | 8695800 |
|  | 8798479 |
|  | 8846784 |
|  | 8917518 |
|  | 9069290 |
|  | 9092573 |
|  | 9130707 |
|  | 9155018 |
|  | 9211903 |
|  | 9278512 |
|  | 9295308 |
|  | 9405468 |
|  | 9465032 |
|  | 9554853 |
|  | 9628874 |
|  | 9753748 |
|  | 9768359 |
|  | 9788880 |
|  | 9792677 |
|  | 9858528 |
|  | 9878398 |
|  | 9889198 |
|  | 12140684 |
|  | 14576193 |
|  | 14588145 |
|  | 15475493 |
|  | 10995439 |
|  | 11116087 |
|  | 16456712 |
|  | 16774848 |
|  | 14574404 |
|  | 15489334 |
|  | 15592455 |
|  | 16532349 |
|  | 16814421 |
|  | 16924234 |
|  | 16927023 |
|  | 16935849 |
|  | 16951426 |
|  | 17000004 |
|  | 17012370 |
|  | 17081983 |
|  | 17085432 |
|  | 17126905 |
|  | 17188240 |
|  | 17192257 |
|  | 17196171 |
|  | 17202865 |
|  | 17207971 |
|  | 17234706 |
|  | 17241234 |
|  | 17244678 |
|  | 17255097 |
|  | 17255949 |
|  | 17277157 |
|  | 17284589 |
|  | 17303382 |
|  | 17303384 |
|  | 17307333 |
|  | 17316568 |
|  | 17332506 |
|  | 17341442 |
|  | 17347580 |
|  | 17373649 |
|  | 17406030 |
|  | 17438131 |
|  | 17446930 |
|  | 17464174 |
|  | 17468755 |
|  | 17519236 |
|  | 17524358 |
|  | 17531530 |
|  | 17543571 |
|  | 17548358 |
|  | 17583675 |
|  | 17612563 |
|  | 17613714 |
|  | 17616668 |
|  | 17624368 |
|  | 17675521 |
|  | 7923354 |
|  | 8910361 |
|  | 9095200 |
|  | 9753691 |
|  | 11593045 |
|  | 12872135 |
|  | 16177070 |
|  | 16413747 |
|  | 16442778 |
|  | 16476765 |
|  | 16514068 |
|  | 16574378 |
|  | 16806764 |
|  | 16844343 |
|  | 16889766 |
|  | 16924238 |
|  | 16937126 |
|  | 16978838 |
|  | 17040971 |
|  | 17117477 |
|  | 17158101 |
|  | 17158963 |
|  | 17167049 |
|  | 17182102 |
|  | 17185377 |
|  | 17187070 |
|  | 17194700 |
|  | 17202326 |
|  | 17210568 |
|  | 17219434 |
|  | 17234967 |
|  | 17244351 |
|  | 17255352 |
|  | 17267699 |
|  | 17277164 |
|  | 17291992 |
|  | 17292829 |
|  | 17304211 |
|  | 17334779 |
|  | 17344394 |
|  | 17346981 |
|  | 17362930 |
|  | 17373651 |
|  | 17441692 |
|  | 17464186 |
|  | 17467682 |
|  | 17468757 |
|  | 17481611 |
|  | 17490618 |
|  | 17534150 |
|  | 17544802 |
|  | 17574041 |
|  | 17588539 |
|  | 17613723 |
|  | 17621549 |
|  | 17658519 |
|  | 17699753 |
|  | 9122194 |
|  | 16983391 |
|  | 17069850 |
|  | 17133350 |
|  | 17151195 |
|  | 17172269 |
|  | 17196178 |
|  | 17208994 |
|  | 17235686 |
|  | 17293487 |
|  | 17308014 |
|  | 17333127 |
|  | 17401436 |
|  | 17467311 |
|  | 17488713 |
|  | 17496214 |
|  | 17583725 |
|  | 17609504 |
|  | 17882791 |

---

|  |  |
| --- | --- |
| Organism | Homo sapiens {Organism urn:agi-taxid:9606} |
|  | Mus musculus {Organism urn:agi-taxid:10090} |
|  | Rattus norvegicus {Organism urn:agi-taxid:10116} |
|  | Homo sapiens |
|  | Mus musculus |
|  | Rattus norvegicus |
|  | Bos taurus |
|  | Danio rerio |
|  | Human |

---

|  |  |
| --- | --- |
| Mouse chromosome position | 17 14.85 cM |
|  | 17 13.5 cM |

---

|  |  |
| --- | --- |
| OMIM ID | 600289 |
|  | 61407 |
|  | OMIM:600289 |

---

|  |  |
| --- | --- |
| Rat chromosome position | 20p12 |

---

|  |  |
| --- | --- |
| Hugo ID | 6876 |
|  | HGNC:6876 |

---

|  |  |
| --- | --- |
| Human chromosome position | 6p21.31 |
|  | 6p21.3-p21.2 |

---

|  |  |
| --- | --- |
| Swiss-Prot Accession | L7RSM2 |
|  | Q16539 |
|  | A0A024RD15 |
|  | B4E0K5 |
|  | Q16539.3 |
|  | P47811 |
|  | Q5U421 |
|  | P47811.3 |
|  | Q56A33 |
|  | G3V617 |
|  | P70618.3 |
|  | A6ZJ92 |
|  | A8K6P4 |
|  | B0LPH0 |
|  | B5TY32 |
|  | O60776 |
|  | Q13083 |
|  | Q14084 |
|  | Q8TDX0 |
|  | B2KF37 |
|  | B2KF38 |
|  | O08666 |
|  | Q3U6R5 |
|  | Q3UZS3 |
|  | Q8C289 |
|  | Q9JLV8 |
|  | Q9QZ80 |
|  | P70618 |
|  | Q15454 |
|  | MK14\_HUMAN |
|  | MK14\_MOUSE |
|  | B2KF37\_MOUSE |
|  | Q56A33\_RAT |

---

|  |  |
| --- | --- |
| PIR ID | S53536 |
|  | I49066 |

---

|  |  |
| --- | --- |
| GenBank ID | NC\_000006 |
|  | XR\_926065 |
|  | NM\_139012 |
|  | NP\_620581 |
|  | NM\_139014 |
|  | NP\_620583 |
|  | NM\_001315 |
|  | NP\_001306 |
|  | XM\_011514310 |
|  | XP\_011512612 |
|  | XM\_017010300 |
|  | XP\_016865789 |
|  | XM\_017010299 |
|  | XP\_016865788 |
|  | XM\_017010301 |
|  | XP\_016865790 |
|  | XM\_017010304 |
|  | XP\_016865793 |
|  | XM\_017010303 |
|  | XP\_016865792 |
|  | XM\_017010302 |
|  | XP\_016865791 |
|  | NM\_139013 |
|  | NP\_620582 |
|  | XM\_006714998 |
|  | XP\_006715061 |
|  | CH471081 |
|  | EAX03867 |
|  | EAX03868 |
|  | EAX03869 |
|  | EAX03870 |
|  | EAX03871 |
|  | EAX03872 |
|  | EAX03873 |
|  | EU332860 |
|  | ABY87549 |
|  | JD591565 |
|  | CIW96073 |
|  | JX512452 |
|  | AGC09599 |
|  | U19775 |
|  | AAC50329 |
|  | Z95152 |
|  | AB074150 |
|  | BAB85654 |
|  | AF100544 |
|  | AAF36770 |
|  | AK291709 |
|  | BAF84398 |
|  | AK303414 |
|  | BAG64467 |
|  | BC000092 |
|  | AAH00092 |
|  | BC031574 |
|  | AAH31574 |
|  | BT006933 |
|  | AAP35579 |
|  | CR536505 |
|  | CAG38743 |
|  | DA438432 |
|  | FJ032367 |
|  | ACI00233 |
|  | FJ032368 |
|  | ACI00234 |
|  | L35263 |
|  | AAA57455 |
|  | L35264 |
|  | AAA57456 |
|  | Z25432 |
|  | CAA80919 |
|  | Q16539 |
|  | NC\_000083 |
|  | NM\_001168514 |
|  | NP\_001161986 |
|  | NM\_011951 |
|  | NP\_036081 |
|  | NM\_001168508 |
|  | NP\_001161980 |
|  | XM\_017317480 |
|  | XP\_017172969 |
|  | NM\_001168513 |
|  | NP\_001161985 |
|  | NT\_187005 |
|  | NM\_001357724 |
|  | AC170998 |
|  | CH466606 |
|  | EDL22584 |
|  | EDL22585 |
|  | EDL22586 |
|  | EDL22587 |
|  | CT009661 |
|  | JD591567 |
|  | CIW96269 |
|  | AF128892 |
|  | AAF34818 |
|  | AF195850 |
|  | AAF06348 |
|  | AI132714 |
|  | AK089059 |
|  | BAC40726 |
|  | AK133684 |
|  | BAE21782 |
|  | AK151348 |
|  | BAE30324 |
|  | AK153025 |
|  | BAE31659 |
|  | AK177608 |
|  | AK178357 |
|  | AK185139 |
|  | AK191704 |
|  | AK192555 |
|  | AK195023 |
|  | AK199780 |
|  | AK201673 |
|  | AK205426 |
|  | AK210155 |
|  | AK210810 |
|  | AK216421 |
|  | AK218544 |
|  | AK219197 |
|  | BC012235 |
|  | AAH12235 |
|  | BC018227 |
|  | BC085303 |
|  | AAH85303 |
|  | CX568915 |
|  | D83073 |
|  | BAA19741 |
|  | U10871 |
|  | AAA20888 |
|  | W66694 |
|  | X65067 |
|  | CAA46200 |
|  | P47811 |
|  | NC\_005119 |
|  | XM\_017601781 |
|  | XP\_017457270 |
|  | XM\_006256142 |
|  | XP\_006256204 |
|  | NM\_031020 |
|  | NP\_112282 |
|  | AC\_000088 |
|  | AABR07044432 |
|  | AABR07044433 |
|  | AABR07044434 |
|  | AAHX01099785 |
|  | CH473988 |
|  | EDL96934 |
|  | EDL96935 |
|  | EDL96936 |
|  | EDL96937 |
|  | AB000783 |
|  | BAF80890 |
|  | AF346293 |
|  | AAK15541 |
|  | BC092193 |
|  | AAH92193 |
|  | U73142 |
|  | AAC71059 |
|  | U91847 |
|  | AAB51285 |
|  | P70618 |
|  | AABR06099920 |
|  | AABR06099918 |
|  | AABR06099919 |
|  | AMYH02013551 |
|  | AC\_000039 |
|  | XR\_926067 |
|  | XR\_926068 |
|  | XM\_011514311 |
|  | XP\_006524368 |
|  | XP\_006524367 |
|  | ABBA01038251 |
|  | AC\_000138 |
|  | ABBA01038250 |
|  | AAHY01131921 |
|  | ABBA01038249 |
|  | CAQ52037 |
|  | CAQ52035 |
|  | CAQ52036 |
|  | CAQ52033 |
|  | CAQ52034 |
|  | XM\_006524305 |
|  | XP\_011512613 |
|  | XM\_006524304 |
|  | XR\_241882 |
|  | NC\_018917 |
|  | NT\_007592 |
|  | NW\_923073 |
|  | NW\_001838980 |
|  | AC\_000049 |
|  | CR598726 |
|  | CR612713 |
|  | Q15454 |
|  | NT\_039649 |
|  | NT\_165782 |
|  | NW\_001030605 |
|  | Q5U421 |
|  | NW\_047597 |
|  | NW\_001084789 |
|  | Q56A33 |
|  | 1621646 |
|  | 1621647 |
|  | 20986515 |
|  | 20986516 |
|  | 6996646 |
|  | 6996647 |
|  | NW\_000124 |
|  | 34852317 |
|  | 1181606 |
|  | 3036773 |
|  | 10092589 |
|  | 3392894 |
|  | 3392895 |
|  | 4503069 |
|  | 4503068 |
|  | 26354195 |
|  | 51465701 |
|  | 2499600 |
|  | 2499601 |
|  | 1136798 |
|  | 1136797 |
|  | NT\_086693 |
|  | CAB08440 |
|  | 603917 |
|  | 603918 |
|  | 51465675 |
|  | 603916 |
|  | 603919 |
|  | 19071869 |
|  | 19071868 |
|  | 50551 |
|  | BG087465 |
|  | 50552 |
|  | 30582705 |
|  | 30582704 |
|  | 405743 |
|  | 405742 |
|  | 7109717 |
|  | 7109716 |
|  | 17390519 |
|  | 1945623 |
|  | 51770238 |
|  | 55154531 |
|  | 55154530 |
|  | 1916984 |
|  | 26354196 |
|  | 12652686 |
|  | 13194758 |
|  | 12652687 |
|  | NT\_039548 |
|  | 15126598 |
|  | 15126597 |
|  | 1916983 |
|  | 1346566 |
|  | 13194757 |
|  | 21594896 |
|  | CAB08439 |
|  | 21594895 |
|  | 6273386 |
|  | 13591927 |
|  | 6273387 |
|  | 13591928 |
|  | 531125 |
|  | NW\_043430 |
|  | 531124 |
|  | 10092590 |
|  | 49168495 |
|  | 49168496 |
|  | 20986513 |
|  | 20986514 |
|  | 20986511 |
|  | 20986512 |
|  | 62089482 |
|  | 81909362 |
|  | 74191494 |
|  | 74225499 |
|  | 74227417 |
|  | 81910272 |
|  | 119624273 |
|  | 119624274 |
|  | 119624275 |
|  | 119624276 |
|  | 119624277 |
|  | 119624278 |
|  | 74706557 |
|  | BE589157 |
|  | BM434141 |
|  | CK981031 |
|  | NC\_007324 |
|  | NW\_001494145 |
|  | XM\_614274 |
|  | XP\_614274 |
|  | 30316123 |
|  | 37589891 |
|  | 9836500 |
|  | AAH44128 |
|  | AB030897 |
|  | AI353982 |
|  | AL929282 |
|  | BAB11807 |
|  | BC044128 |
|  | BC059417 |
|  | BE693168 |
|  | NC\_007119 |
|  | NM\_131722 |
|  | NP\_571797 |
|  | NW\_001513952 |
|  | Q9DGE2 |
|  | AW595028 |
|  | 119624272 |
|  | 148690637 |
|  | 148690638 |
|  | 148690639 |
|  | 148690640 |
|  | 149043483 |
|  | 149043484 |
|  | 149043485 |
|  | 149043486 |
|  | O60776 |

---

|  |  |
| --- | --- |
| Swiss-Prot ID | MK14\_HUMAN |
|  | MK14\_MOUSE |
|  | Q56A33\_RAT |
|  | Q5U421\_MOUSE |
|  | B2KF37\_MOUSE |

---

|  |  |
| --- | --- |
| Cell Localization | Cytoplasm |
|  | Nucleus |

---

|  |  |
| --- | --- |
| Ensembl ID | ENSG00000112062 |
|  | ENSP00000229795.3 |
|  | ENST00000229795.7 |
|  | ENSP00000229794.4 |
|  | ENST00000229794.8 |
|  | ENSP00000308669.4 |
|  | ENST00000310795.8 |
|  | ENSP00000419837.1 |
|  | ENST00000468133.5 |
|  | ENSMUSG00000053436 |
|  | ENSMUSP00000004990.6 |
|  | ENSMUST00000004990.13 |
|  | ENSMUSP00000110400.1 |
|  | ENSMUST00000114752.2 |
|  | ENSMUSP00000110402.1 |
|  | ENSMUST00000114754.7 |
|  | ENSMUSP00000061958.8 |
|  | ENSMUST00000062694.15 |
|  | ENSRNOG00000000513 |
|  | ENSRNOP00000000617.3 |
|  | ENSRNOT00000000617.8 |
|  | ENSRNOP00000000618.2 |
|  | ENSRNOT00000000618.6 |
|  | ENSMUSP00000061958 |
|  | ENST00000310795 |
|  | ENSMUST00000062694.14 |
|  | ENSMUST00000004990.12 |
|  | ENSP00000308669 |
|  | ENSMUST00000004990 |
|  | ENST00000229794 |
|  | ENSMUSP00000004990 |
|  | ENSMUSP00000110400 |
|  | ENSP00000229794 |
|  | ENSMUSP00000110402 |
|  | ENSP00000419141 |
|  | ENSRNOT00000000617 |
|  | ENSP00000229795 |
|  | ENSRNOT00000000618 |
|  | ENST00000229795 |
|  | ENST00000468133 |
|  | ENSMUST00000114754 |
|  | ENSMUST00000114752 |
|  | ENSP00000419837 |
|  | ENSRNOP00000000618 |
|  | ENSRNOP00000000617 |
|  | ENSMUST00000062694 |
|  | ENST00000472333 |

---

|  |  |
| --- | --- |
| MGI ID | MGI:1346865 |
|  | 1346865 |
|  | 1338028 |

---

|  |  |
| --- | --- |
| RGD ID | 70496 |
|  | 61407 |

---

|  |  |
| --- | --- |
| Unigene ID | Mm.311337 |
|  | Rn.88085 |
|  | Hs.485233 |
|  | Hs.588289 |
|  | Mm.4437 |
|  | Mm.347796 |
|  | Hs.79107 |
|  | Bt.11377 |
|  | Dr.72252 |
|  | Hs.719314 |
|  | Hs.722031 |

---

|  |  |
| --- | --- |
| Homologene ID | 31777 |

---

|  |  |
| --- | --- |
| Shape | Sickle-vertex |

---

|  |  |
| --- | --- |
| IPI ID | IPI00816843 |
|  | IPI00221141 |
|  | IPI00331732 |
|  | IPI00828805 |
|  | IPI00221143 |
|  | IPI00221142 |
|  | IPI00002857 |
|  | IPI00112346 |
|  | IPI00230807 |
|  | IPI00830415 |
|  | IPI00190530 |
|  | IPI00829435 |
|  | IPI00558535 |

---

|  |  |
| --- | --- |
| Microarray ID | 99978\_s\_at |
|  | 1367697\_at |
|  | U73142\_at |
|  | U73142\_g\_at |
|  | U91847\_s\_at |
|  | rc\_aa924542\_s\_at |
|  | rc\_ai137862\_s\_at |
|  | rc\_ai171630\_s\_at |
|  | A\_43\_P12479 |
|  | NM\_031020 |
|  | 1416703\_at |
|  | 1416704\_at |
|  | 1426104\_at |
|  | 1451927\_a\_at |
|  | u10871\_s\_at |
|  | Msa.8386.0\_s\_at |
|  | Msa.343.0\_at |
|  | Msa.8386.0\_at |
|  | A\_65\_P02959 |
|  | L0241G04-3 |
|  | A\_51\_P325343 |
|  | NM\_011951 |
|  | A\_65\_P19663 |
|  | AF195850.1 |
|  | L35263 |
|  | 37733\_at |
|  | 78240\_at |
|  | 1671\_s\_at |
|  | 202530\_at |
|  | 210449\_x\_at |
|  | 211087\_x\_at |
|  | 211561\_x\_at |
|  | 1999\_s\_at |
|  | L35263\_at |
|  | L35253\_s\_at |
|  | A\_23\_P214696 |
|  | I\_957350 |
|  | A\_23\_P426292 |
|  | A\_14\_P139376 |
|  | A\_14\_P138042 |
|  | A\_14\_P129108 |
|  | A\_14\_P111428 |
|  | A\_24\_P283288 |
|  | A\_24\_P397566 |
|  | 704 |
|  | OH7873 |
|  | M190 |
|  | R519 |
|  | OR0693 |

---

|  |  |
| --- | --- |
| Homo sapiens Chromosome position | 6p21.3-p21.2 |

---

|  |  |
| --- | --- |
| Ensembl Transcript ID | ENST00000229794 |
|  | ENST00000229795 |
|  | ENST00000310795 |

---

|  |  |
| --- | --- |
| ENSEMBL | ENSG00000112062 |

---

|  |  |
| --- | --- |
| FunctionalClass | MAP kinase |
|  | MAP kinase kinase |
|  | MP kinase |
|  | kinase |
|  | protein C-terminus binding |
|  | protein binding |
|  | protein kinase |
|  | protein-tyrosine kinase |
|  | ATP binding |
|  | nucleotide binding |
|  | protein serine/threonine kinase |
|  | transferase |
|  | cAMP-dependent protein kinase |
|  | protein kinase CK2 |

---

|  |  |
| --- | --- |
| Hugo Symbol | MAPK14 |

---

|  |  |
| --- | --- |
| Bos taurus Chromosome position | 23 |

---

|  |  |
| --- | --- |
| Mus musculus Chromosome position | 17 13.5 cM |

---

|  |  |
| --- | --- |
| KEGG pathway | Toll-like receptor signaling pathway |
|  | MAPK signaling pathway |
|  | Fc epsilon RI signaling pathway |
|  | GnRH signaling pathway |
|  | Leukocyte transendothelial migration |
|  | VEGF signaling pathway |

---

|  |  |
| --- | --- |
| ProteinAtlas SubcellularLocation | Main location: Vesicles; Other location: Nucleus but not nucleoli;Cytoplasm;Golgi apparatus; Expression type: Staining; Reliability: Uncertain |

---

|  |  |
| --- | --- |
| State | isoform 1 |
|  | isoform 2 |
|  | isoform 3 |
|  | isoform 4 |
|  | p |

---

|  |  |
| --- | --- |
| Rattus norvegicus Chromosome position | 20p12 |

---

|  |  |
| --- | --- |
| Ensembl Gene ID | ENSG00000112062 |

---

|  |  |
| --- | --- |
| Keyword | map kinase |
|  | ERK activator kinase |
|  | tumor necrosis factor |
|  | SAP kinase |
|  | MAP kinase kinase kinase |
|  | phospholipase C |
|  | map kinases |

---

|  |  |
| --- | --- |
| ProteinAtlas NormalTissue | Tissue: liver; Cell type: hepatocytes; Level: Low; Expression type: APE; Reliability: Low |
|  | Tissue: lung; Cell type: pneumocytes; Level: Low; Expression type: APE; Reliability: Low |
|  | Tissue: adrenal gland; Cell type: glandular cells; Level: Medium; Expression type: APE; Reliability: Low |
|  | Tissue: appendix; Cell type: glandular cells; Level: Medium; Expression type: APE; Reliability: Low |
|  | Tissue: appendix; Cell type: lymphoid tissue; Level: Low; Expression type: APE; Reliability: Low |
|  | Tissue: bone marrow; Cell type: hematopoietic cells; Level: High; Expression type: APE; Reliability: Low |
|  | Tissue: breast; Cell type: glandular cells; Level: Low; Expression type: APE; Reliability: Low |
|  | Tissue: bronchus; Cell type: respiratory epithelial cells; Level: Medium; Expression type: APE; Reliability: Low |
|  | Tissue: cerebellum; Cell type: cells in granular layer; Level: Low; Expression type: APE; Reliability: Low |
|  | Tissue: cerebellum; Cell type: cells in molecular layer; Level: None; Expression type: APE; Reliability: Low |
|  | Tissue: cerebellum; Cell type: Purkinje cells; Level: Low; Expression type: APE; Reliability: Low |
|  | Tissue: cerebral cortex; Cell type: glial cells; Level: Low; Expression type: APE; Reliability: Low |
|  | Tissue: cerebral cortex; Cell type: neuronal cells; Level: Medium; Expression type: APE; Reliability: Low |
|  | Tissue: cervix, uterine; Cell type: glandular cells; Level: Low; Expression type: APE; Reliability: Low |
|  | Tissue: cervix, uterine; Cell type: squamous epithelial cells; Level: Low; Expression type: APE; Reliability: Low |
|  | Tissue: colon; Cell type: glandular cells; Level: Medium; Expression type: APE; Reliability: Low |
|  | Tissue: duodenum; Cell type: glandular cells; Level: Medium; Expression type: APE; Reliability: Low |
|  | Tissue: epididymis; Cell type: glandular cells; Level: Medium; Expression type: APE; Reliability: Low |
|  | Tissue: esophagus; Cell type: squamous epithelial cells; Level: Low; Expression type: APE; Reliability: Low |
|  | Tissue: fallopian tube; Cell type: glandular cells; Level: Medium; Expression type: APE; Reliability: Low |
|  | Tissue: gall bladder; Cell type: glandular cells; Level: Medium; Expression type: APE; Reliability: Low |
|  | Tissue: heart muscle; Cell type: myocytes; Level: Low; Expression type: APE; Reliability: Low |
|  | Tissue: hippocampus; Cell type: glial cells; Level: Low; Expression type: APE; Reliability: Low |
|  | Tissue: hippocampus; Cell type: neuronal cells; Level: Medium; Expression type: APE; Reliability: Low |
|  | Tissue: kidney; Cell type: cells in glomeruli; Level: Low; Expression type: APE; Reliability: Low |
|  | Tissue: kidney; Cell type: cells in tubules; Level: Medium; Expression type: APE; Reliability: Low |
|  | Tissue: lateral ventricle; Cell type: glial cells; Level: Low; Expression type: APE; Reliability: Low |
|  | Tissue: lateral ventricle; Cell type: neuronal cells; Level: Medium; Expression type: APE; Reliability: Low |
|  | Tissue: liver; Cell type: bile duct cells; Level: Low; Expression type: APE; Reliability: Low |
|  | Tissue: lung; Cell type: macrophages; Level: Medium; Expression type: APE; Reliability: Low |
|  | Tissue: lymph node; Cell type: germinal center cells; Level: Low; Expression type: APE; Reliability: Low |
|  | Tissue: lymph node; Cell type: non-germinal center cells; Level: Medium; Expression type: APE; Reliability: Low |
|  | Tissue: nasopharynx; Cell type: respiratory epithelial cells; Level: Medium; Expression type: APE; Reliability: Low |
|  | Tissue: oral mucosa; Cell type: squamous epithelial cells; Level: Low; Expression type: APE; Reliability: Low |
|  | Tissue: ovary; Cell type: follicle cells; Level: Low; Expression type: APE; Reliability: Low |
|  | Tissue: ovary; Cell type: ovarian stroma cells; Level: Low; Expression type: APE; Reliability: Low |
|  | Tissue: pancreas; Cell type: exocrine glandular cells; Level: Low; Expression type: APE; Reliability: Low |
|  | Tissue: pancreas; Cell type: islets of Langerhans; Level: Low; Expression type: APE; Reliability: Low |
|  | Tissue: parathyroid gland; Cell type: glandular cells; Level: Low; Expression type: APE; Reliability: Low |
|  | Tissue: placenta; Cell type: decidual cells; Level: Medium; Expression type: APE; Reliability: Low |
|  | Tissue: placenta; Cell type: trophoblastic cells; Level: Medium; Expression type: APE; Reliability: Low |
|  | Tissue: prostate; Cell type: glandular cells; Level: Low; Expression type: APE; Reliability: Low |
|  | Tissue: rectum; Cell type: glandular cells; Level: Medium; Expression type: APE; Reliability: Low |
|  | Tissue: salivary gland; Cell type: glandular cells; Level: Low; Expression type: APE; Reliability: Low |
|  | Tissue: seminal vesicle; Cell type: glandular cells; Level: Low; Expression type: APE; Reliability: Low |
|  | Tissue: skeletal muscle; Cell type: myocytes; Level: Medium; Expression type: APE; Reliability: Low |
|  | Tissue: skin; Cell type: epidermal cells; Level: Low; Expression type: APE; Reliability: Low |
|  | Tissue: small intestine; Cell type: glandular cells; Level: Medium; Expression type: APE; Reliability: Low |
|  | Tissue: smooth muscle; Cell type: smooth muscle cells; Level: Low; Expression type: APE; Reliability: Low |
|  | Tissue: spleen; Cell type: cells in red pulp; Level: Medium; Expression type: APE; Reliability: Low |
|  | Tissue: spleen; Cell type: cells in white pulp; Level: Low; Expression type: APE; Reliability: Low |
|  | Tissue: stomach, lower; Cell type: glandular cells; Level: Medium; Expression type: APE; Reliability: Low |
|  | Tissue: stomach, upper; Cell type: glandular cells; Level: Medium; Expression type: APE; Reliability: Low |
|  | Tissue: testis; Cell type: cells in seminiferus ducts; Level: Medium; Expression type: APE; Reliability: Low |
|  | Tissue: testis; Cell type: Leydig cells; Level: Low; Expression type: APE; Reliability: Low |
|  | Tissue: thyroid gland; Cell type: glandular cells; Level: Medium; Expression type: APE; Reliability: Low |
|  | Tissue: tonsil; Cell type: germinal center cells; Level: Low; Expression type: APE; Reliability: Low |
|  | Tissue: tonsil; Cell type: non-germinal center cells; Level: Medium; Expression type: APE; Reliability: Low |
|  | Tissue: tonsil; Cell type: squamous epithelial cells; Level: Medium; Expression type: APE; Reliability: Low |
|  | Tissue: urinary bladder; Cell type: urothelial cells; Level: Medium; Expression type: APE; Reliability: Low |
|  | Tissue: uterus, post-menopause; Cell type: cells in endometrial stroma; Level: None; Expression type: APE; Reliability: Low |
|  | Tissue: uterus, post-menopause; Cell type: glandular cells; Level: Medium; Expression type: APE; Reliability: Low |
|  | Tissue: uterus, pre-menopause; Cell type: cells in endometrial stroma; Level: Low; Expression type: APE; Reliability: Low |
|  | Tissue: uterus, pre-menopause; Cell type: glandular cells; Level: Low; Expression type: APE; Reliability: Low |
|  | Tissue: vagina; Cell type: squamous epithelial cells; Level: Low; Expression type: APE; Reliability: Low |
|  | Tissue: vulva/anal skin; Cell type: epidermal cells; Level: Low; Expression type: APE; Reliability: Low |

---

|  |  |
| --- | --- |
| State Description | Transcript Variant: This variant (1) encodes the longest isoform (1). |
|  | Transcript Variant: This variant (2) contains a different segment within the coding region when compared to variant 1. The translation frame remains the same, and the resulting isoform 2 has an internal segment different from that of isoform 1. |
|  | Transcript Variant: This variant (3) contains a different internal segment within the coding region, and a different 3' coding region as well as a different 3' UTR, when compared to variant 1. It thus encodes an isoform that has a different internal se... |
|  | Transcript Variant: This variant (4) contains a different internal segment when compared to variant 1. It thus encodes an isoform that has a different and shorter internal segment, as compared to isoform 1. |

---

|  |  |
| --- | --- |
| EnzymeConsortium | 2.7.1.37 |

---
